# Parental management of autoimmune disease with complementary and alternative medicine: a scoping review of the literature in OECD countries

**DOI:** 10.1186/s12906-025-04929-4

**Published:** 2025-07-10

**Authors:** Jordana Maio, Caroline A. Smith, Paul R. Ward

**Affiliations:** 1 Torrens University, Adelaide, Australia; 2 Western Sydney University, Sydney, Australia

**Keywords:** Autoimmune disease, Children, Complementary and alternative medicine, Health service use

## Abstract

**Introduction:**

The prevalence of autoimmune disease (AD) is increasing in both paediatric and adult populations, resulting in a rise in healthcare utilisation for symptom management. With no known cure for ADs, management options include conventional medical treatment and/or complementary and alternative medicine (CAM) approaches. Despite the high cost of CAM therapy in Australia, its use continues to rise, especially among adults and children with chronic disease.

**Methods:**

This review was guided by the JBI methodology for scoping reviews. We reported using the Preferred Reporting Items for Systematic Reviews and Meta-Analyses extension for Scoping Reviews (PRISMA-ScR) checklist. Database searched included OVID (Medline, Embase, PsycInfo) CINAHL, Scopus, Web of Science, ProQuest, and Google Scholar. Only primary empirical papers were included. Screening and data extraction were conducted by two reviewers independently with a third reviewer resolving discrepancies.

**Results:**

Our review identified 42 primary research papers published between 1990 and 2021 that addressed parental management of AD with CAM. Commonly reported CAM practices included massage, homeopathy, chiropractic care, and acupuncture, with vitamins and minerals being the most frequently mentioned CAM products. Parents cited dissatisfaction with conventional medication, concerns about its side effects, and the perception of CAM as natural or safer than conventional medicine as primary reasons for CAM use. Parental CAM use strongly predicted child CAM use, yet there was low disclosure of CAM practices to conventional physicians. Reasons for non-disclosure included concerns about negative responses from physicians and perceptions of limited physician understanding of CAM. Parental educational level and family income were also predictive of CAM use.

**Conclusions:**

This review highlights the widespread use of CAM by parents managing their children's AD and emphasises the need for improved communication between parents and healthcare providers. Methodological inconsistencies highlight the necessity for standardised protocols in future CAM research. Additionally, future studies should recognise the interplay between social structures and individual agency in shaping healthcare decisions.

**Clinical trial number:**

Not applicable.

**Protocol registration DOI:**

https://doi.org/10.17605/OSF.IO/9NJCE.

**Supplementary Information:**

The online version contains supplementary material available at 10.1186/s12906-025-04929-4.

## Introduction

### Autoimmune diseases in childhood

Once considered rare, autoimmune diseases (AD) are becoming increasingly common among adults and children [1, 2]. In fact, ADs are now one of the fastest growing chronic conditions in Australia, with five percent of the Australian population currently diagnosed with an AD [3]. Globally, the prevalence of ADs is around three to five percent, and more specifically, five percent in paediatric age [4, 5]. Over the past 30 years, there has been a significant rise in the frequency of ADs, with a net yearly increase of three to seven percent worldwide [6]. Recent studies suggest this trend may increase in the aftermath of COVID-19, as mounting evidence indicates an elevated incidence of ADs following infection with the virus [7–9]. As the prevalence and incidence of ADs continue to increase, there is a corresponding rise in health service utilisation, for diagnosis and management [10].

The exact aetiology of AD is unknown, however contemporary theories suggest a genetic disposition and environmental factors (such as an infection or a toxin) may trigger its onset [1]. ADs can occur at any age, however different ADs have their own characteristic age of onset [1]. Among children, the most common ADs include Hashimoto's and Graves disease, Coeliac disease, type 1 diabetes Mellitus (T1DM), and juvenile idiopathic arthritis (JIA) [3–5]. Currently, no tools exist to predict a person’s risk of developing an AD [1], impacting the development of preventive measures or treatments.

There are no known cures for ADs [3], therefore management of symptoms is vital to enhance the quality of life for individuals with AD. Treatment options often depend on the stage and type of AD [3]. Complementary and alternative medicine (CAM) has been gaining popularity in Australia and worldwide as an approach to manage chronic disease [11–13].

### Complementary and alternative medicine use for children

CAM is a modality that has long offered alternative treatment to conventional medical care. While no consensus exists in the literature regarding a common definition [11, 14, 15], CAM is generally described as an umbrella term encompassing a diverse range of healthcare practices that fall outside the conventional medical system [11, 14, 16, 17]. The term complementary refers to a non-conventional approach used in conjunction with conventional medicine, whereas alternative refers to a non-conventional approach used in place of conventional medicine [18].

In Australia, and globally, there has been an increase in CAM use, most notably in adults and children with chronic disease or comorbidities [13, 14, 19–21]. A 2018 study investigating the prevalence of CAM use in Australia, reported over two thirds of parents are using CAM for their children under 18 years of age; an increase of over 20% from Australian studies reported over a decade ago [11, 13, 22]. The most commonly consulted CAM practitioners for Australian children include naturopaths/herbalists, chiropractors, osteopaths, homeopaths, traditional Chinese practitioners, nutritionists and massage therapists; whilst the most frequently used CAM products include vitamins and minerals, herbal medicine, essential oils and homeopathic medicines [13]. Interestingly, certain regions in Australia exhibit a higher number of CAM practitioners compared to conventional medical practitioners [17]. This trend is particularly prominent in rural areas, where the utilisation of CAM is attributed to factors such as limited availability of conventional healthcare services and dissatisfaction of care from mainstream providers [23].

Given the increasing prevalence and interest in CAM, it is notable that the earliest documented reference to CAM use in children with AD appeared as an abstract published in 1984 by Hoyeraal et al. [24]. The earliest full-text paper included in this review [25] dates from 1990. Studies that focussed on parental use of CAM therapies assert CAM is favoured by parents in general paediatric populations due to its perceived "natural" qualities, which are often associated with safety and trust in the system [19, 26]. Additionally, mothers of children with chronic diseases tend to explore CAM therapies when conventional treatment options are limited or when their children do not respond well to or experience side effects from conventional medical treatments [27, 28]. Many studies have found that at least half of CAM users do not disclose their CAM use to their conventional physicians citing reasons such as fear of conflict, negative responses, and past unpleasant experiences [11, 14, 23, 27–29]. While the majority of research in this area has focused on general paediatric populations, there is a growing body of literature specifically exploring CAM use in children with chronic diseases [27, 28].

### Justification for this review

The majority of papers that focus on CAM use for children with ADs are cross sectional in design, with a paucity of quality review papers [15, 30–35]. While useful in identifying literature in this field, the majority of reviews in this area exhibit variable methodological quality with no systematic search or synthesis [15, 30–35]. Ferro and Speechley [36] appear to be the only researchers who have conducted a systematic review in this area, although this was specifically investigating the prevalence and evidence of CAM use in Juvenile Idiopathic Arthritis (JIA) as opposed to across a number of ADs. Given the increasing prevalence of ADs in children and the subsequent rise in healthcare utilisation, this review will be the first scoping review that aims to comprehensively assess all empirical research investigating the parental management of AD with CAM, providing a solid and high-quality foundation to guide future research.

### Objectives of this review

The objective of this scoping review is to identify and summarise the current state of knowledge regarding parental management of AD with CAM. The specific objectives are as follows:

1. Characterise the types of CAM used by parents for their child with an AD
2. Describe why parents choose CAM for their child with an AD
3. Describe the experiences of parents using CAM for their child with an AD

## Methods

A scoping review protocol was published and registered with Open Science Framework (DOI10.17605/OSF.IO/5WUM6). The scoping review was conducted in accordance with the JBI methodology for scoping reviews [37] and reported in line with the Preferred Reporting Items for Systematic Reviews and Meta-Analyses extension for Scoping Reviews (PRISMA-ScR) [38].

### Inclusion criteria

Eligibility criteria were defined through expansion of the PCC (population, concept and context) formula as recommended by JBI and detailed below.

#### Population

Participants included primary care givers of children (0 to 18 years) who have been clinically diagnosed with an AD and have engaged in at least one form of complementary or alternative therapy approach (CAM approaches detailed in CAM search criteria).

Exclusion criteria included primary care givers only seeking allopathic management of their child’s AD and primary carers seeking alternative treatment for children over the age of 18.

#### Concept

The concept was defined as the use of CAM for AD management. The CAM approaches included in this review included those used in the search string of Reid et al. ‘s [14] systematic review of complementary therapy use in Australia. The terms “integrative medicine” and “integrative health” were added to the search string, reflecting their emergence in the literature as terms encompassing the integration of conventional and complementary medicine. This addition aimed to capture comprehensive coverage of literature exploring the intersection of healthcare modalities in AD management (Additional file 5).

Similar to CAM, there is no consensus in the literature on a common definition of AD. Despite proposals from multiple researchers, no widely accepted set of criteria exist to define AD [2]. This review included ADs that are listed on the Autoimmune Association website [39].

#### Context

This scoping review considered studies that included countries as part of the Organisation for Economic Co-operation and Development (OECD) that meet the World Bank definition of high income for fiscal year 2023. This included countries with a gross national income per capita of US$13,206 or more [40]. This excluded 5 OECD members not on the World Bank list: Columbia, Costa Rica, Korea, Mexico and Turkey. These countries were selected to better align with the Australian context, ensuring relevance to healthcare practices and socioeconomic factors pertinent to the scope of this review.

The inclusion and exclusion criteria for this study were developed based on the research question and were defined before the screening process to ensure consistency in decision-making. Please see the inclusion and exclusion criteria further detailed in Table 1.

**Table 1:** Inclusion and Exclusion Criteria

Inclusion Criteria	Exclusion Criteria
• Type of publication: published peer-reviewed journal articles with available full text • Timeframe: all full-text articles retrieved in database searches (with no time limits applied) • Type of study: qualitative, quantitative, cross sectional, mixed methods, case reports or case-controlled series • Language: English • Population: primary caregivers of 0–18-year-olds • Location: developed countries that are part of the OECD that meet the world bank definition of high income for fiscal year 2023	• Studies focusing on children using only conventional medical treatments for AD • Studies involving primary caregivers of children older than 18 years (rationale: anyone over the age of 18 is considered an adult in Australia and can make their own decisions based on their healthcare) • Commentaries, reviews, opinion papers, editorials, grey literature

### Search strategy

The search strategy incorporated all identified concepts and keywords detailed in Additional file 5. Guidance and feedback from the University librarian informed the selection of keywords and facilitated the adaptation of the search across various databases. The final search was conducted on 8 th March 2025 with publication parameters set to include studies published up to 31 st December 2024. Databases searched, included MEDLINE (Ovid), Embase (Ovid), CINAHL (EBSCOhost), PsycINFO (Ovid), Scopus, ProQuest, Web of Science, and Google Scholar. The reference lists of all studies selected for critical appraisal were screened for additional studies.

###  Study/source of evidence selection

**Fig. 1:**
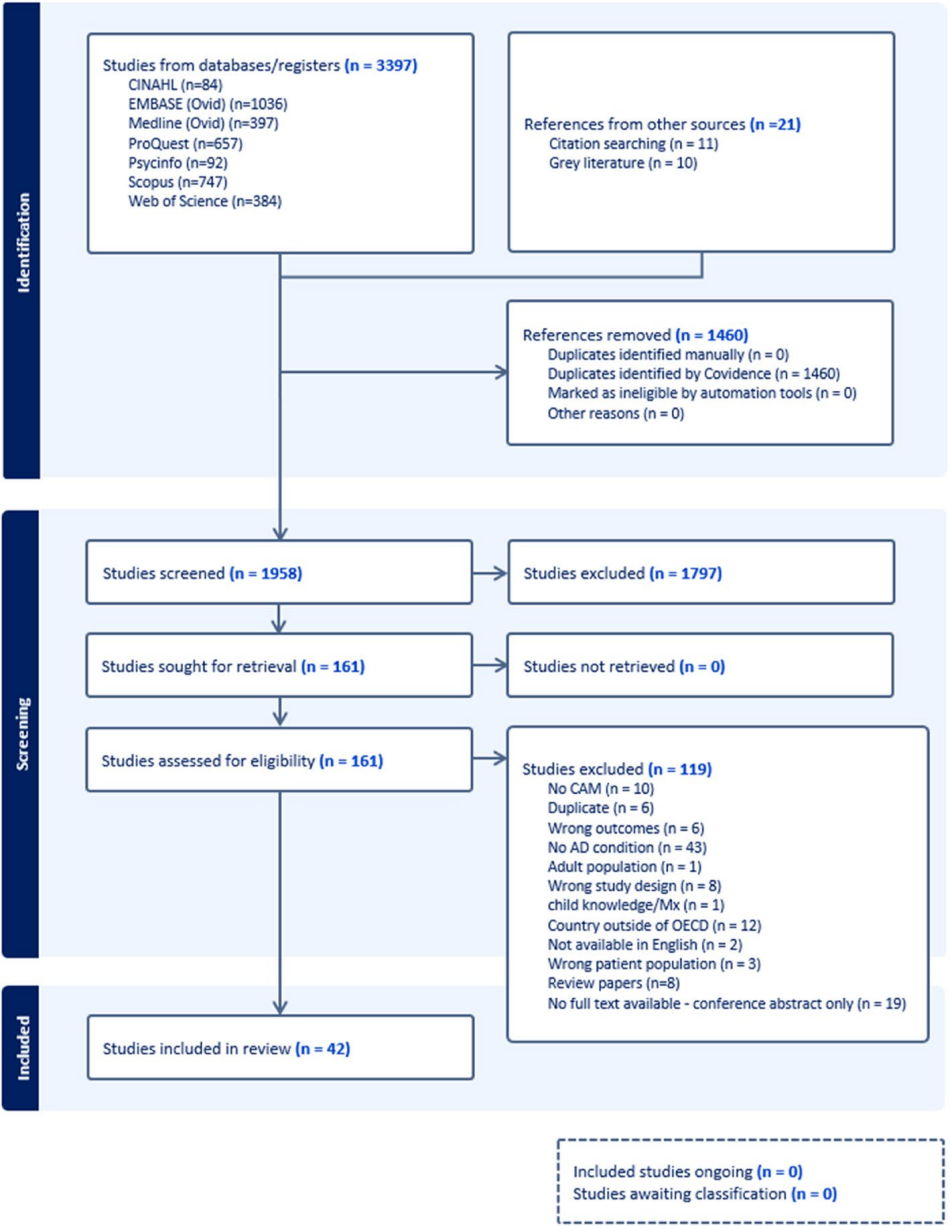
PRISMA Flow Diagram

Following the search, all identified citations were collated and uploaded into EndNote v.X20:0.5/2023 (Clarivate Analytics, PA, USA) and duplicates removed. Titles and abstracts were screened using Covidence (Veritas Health Innovation, Melbourne, Australia), by two or more independent reviewers for assessment against the inclusion. The full text of selected citations was assessed in detail against the inclusion criteria by two or more independent reviewers. Any disagreements that arose between the reviewers at each stage of the selection process were resolved through consultation with a third reviewer. Reasons for exclusion of sources at full text that didn’t meet the inclusion criteria were recorded and reported in the PRISMA diagram (Fig. 1).

### Data extraction

Following full-text review, data were extracted by two or more independent reviewers using a customised data extraction table, which was collaboratively developed and piloted by the authors. Data relating to the following characteristics were extracted: Year of publication, lead author, country in which the study was conducted, aim of the study, study design, who the research was about, who completed surveys or answered questions, types of AD diagnoses, study inclusion and exclusion criteria, number of participants, self identified reasons for using CAM, reasons for not using CAM, perceived benefits/efficacy, experiences of parents using CAM, and types of CAM used. Characteristics of respondents were captured, including carer type and age, if the respondent had used CAM before, information regarding where parents source information about CAM and whether physicians were aware of its use. Socio-demographic and cultural information was captured, including parental education, income, ethnicity, health insurance status and whether they were city or country dwellers. Finally, characteristics of patients were also recorded, including children’s age, sex, disease severity, overall health status, disease duration or time since diagnosis, number of CAM therapies used, conventional medical use and comorbidities. One researcher extracted each of the articles and completed the extraction sheet.

### Data analysis

A descriptive analysis of the included studies was undertaken, focusing on key study characteristics including year of publication, lead author, country of study, research design, sample size, data collection period, types of AD, and CAM modalities used. Additionally, reasons reported for CAM utilisation were categorised descriptively into themes reflecting human agency and social structural factors. Parental experiences associated with CAM usage, including perceived benefits, reported efficacy, adverse events, and reasons for non-utilisation, were also summarised descriptively.

## Results

After merging search results and removing duplicates, a total of 1958 abstracts were identified, with 161 articles retained for full-text screening. From the full-text review, 42 articles were included in the scoping review (see Fig. 1). Additional files 1–4 provide information about the included articles.

### Characteristics of included articles

The research papers used various terms to refer to CAM. These included Complementary and Alternative Medicine (*n* = 25), Complementary/Alternative Medicine (*n* = 2), Complementary Medicine (*n* = 2), Unconventional Remedies, Complementary and Alternative Therapies, Complementary and Alternative Health Care, and Integrative Medicine (*n* = 1).

**Fig. 2:**
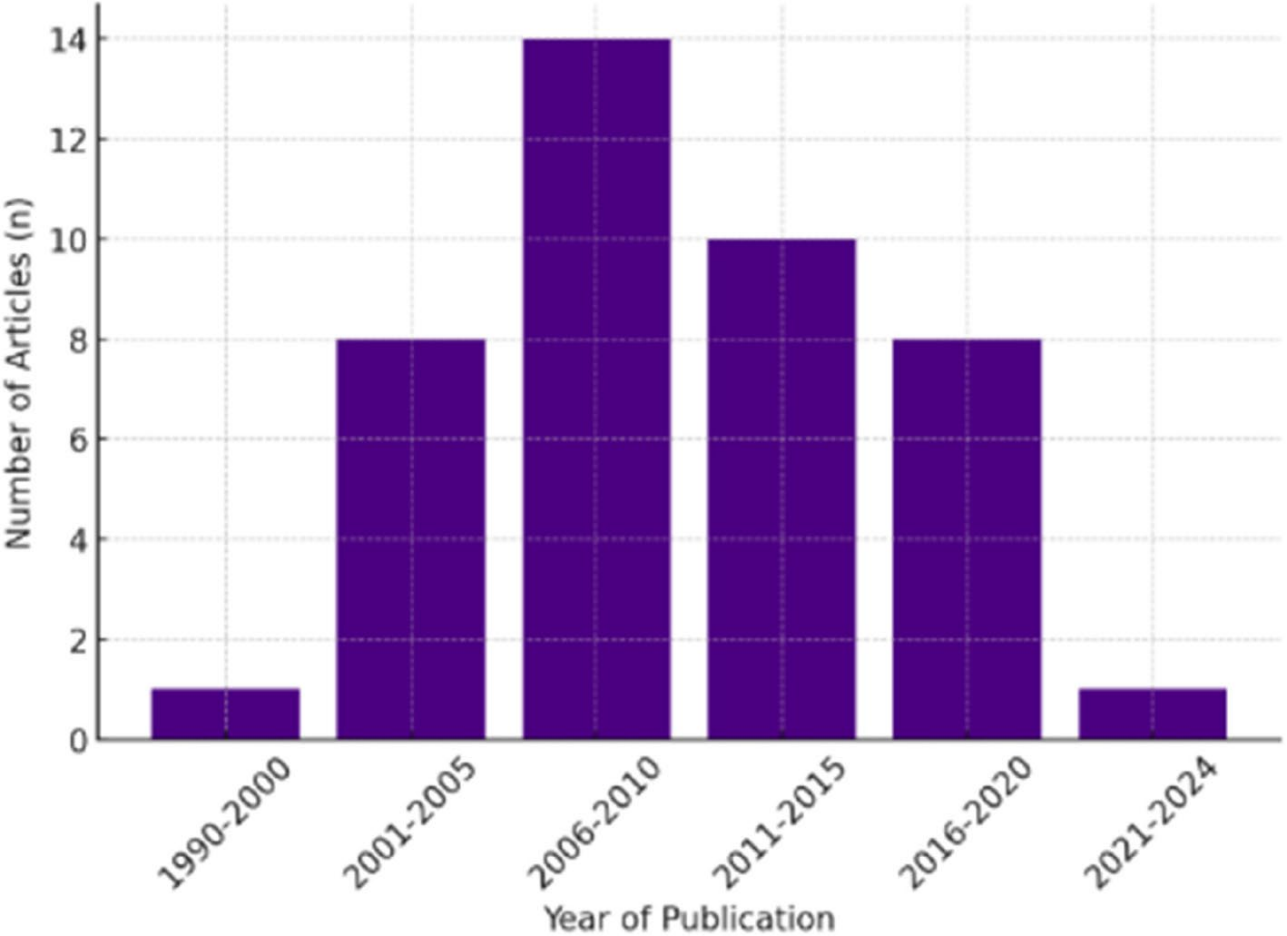
Year of publication of included articles from 1990

Southwood et al.’s [25] seminal paper stands as the earliest paper that met this study’s inclusion criteria, providing insights into paediatric AD and complementary therapies. Interestingly, an eleven year gap lapsed before any papers were published following Southwood et al.’s [25] article. Notably, a peak in publications took place between 2006–2010 (refer to Fig. 2, *n* = 14, 33%).

**Fig. 3:**
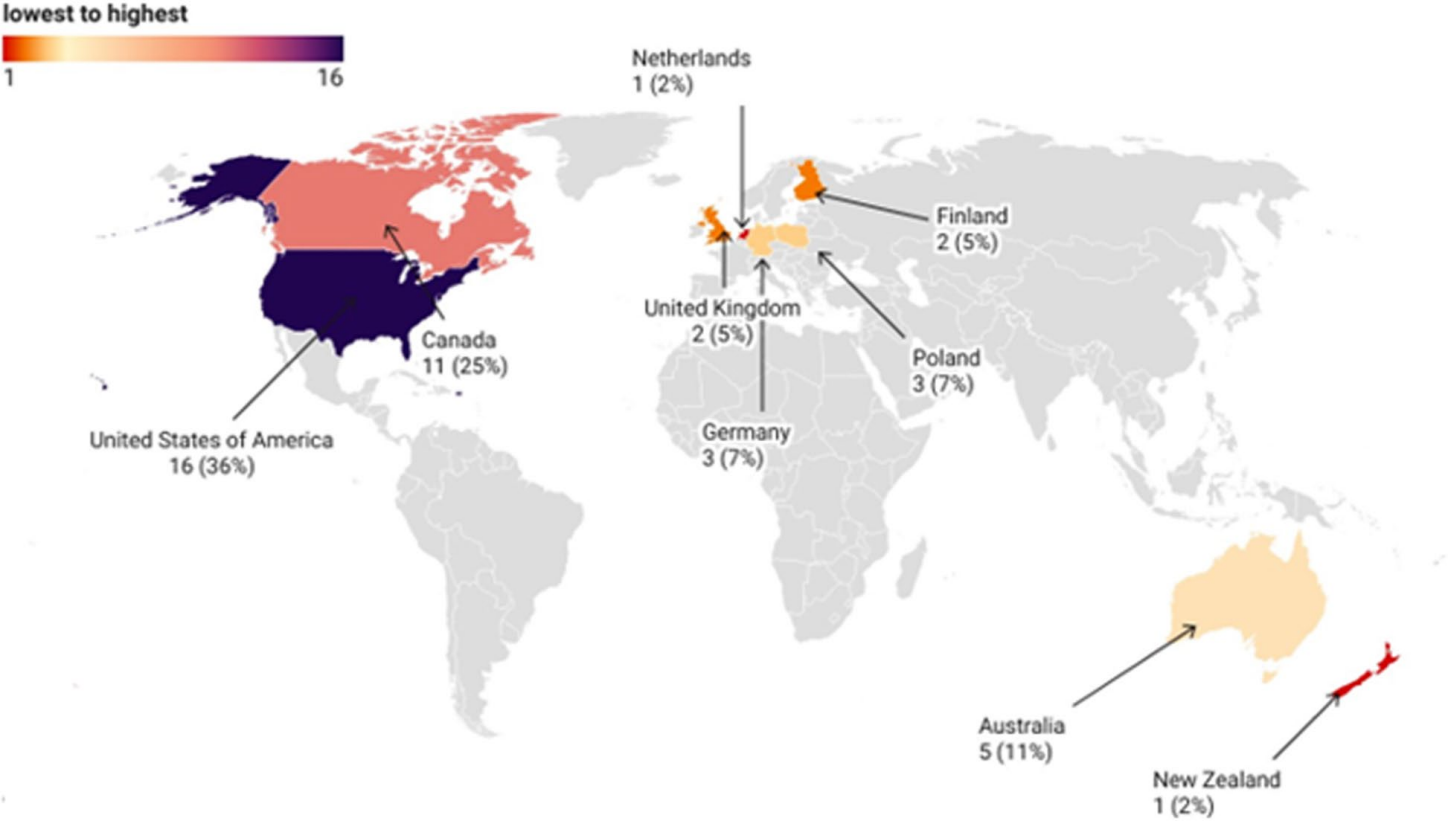
Choropleth of document distribution by geographic region

The United States contributed the highest number of publications (*n* = 16, 36%), followed by Canada (*n* = 11, 25%), Australia (*n* = 5, 11%), Germany and Poland (*n* = 3, 7%), the United Kingdom and Finland (*n* = 2, 5%), and the Netherlands and New Zealand, each with *n* = 1, 2% (refer to Table 2 and Fig. 3).

**Table 2:** Characteristics of included studies (*n* = 42)

Lead author, Year of Publication, Country	Study Design	Types of AD	Sample Size	Data Collection Period
Adams (2013) Canada [41]	Cross Sectional	Crohn's disease, coeliac disease	926	Unspecified
Adams (2014) Canada [42]	Cross Sectional	Crohn's disease, coeliac disease, rheumatoid arthritis, diabetes	215	Unspecified
April (2009) Canada [43]	Cross Sectional	Juvenile Idiopathic Arthritis (JIA)	182	Unspecified
Ball (2005) United States of America [44]	Cross Sectional	Type 1 Diabetes Mellitus (T1DM), rheumatological disorders	505	February—August, 2001
Barczykowska (2008) Poland [45]	Cross Sectional	JIA	30	Unspecified
Bode (2001) Germany [46]	Cross Sectional	Diabetes	252	April to July 1998
Ceballos (2014) United States of America [47]	Cross Sectional	Crohn's disease, Ulcerative colitis	160	Unspecified
Cotton (2010) United States of America [48]	Cross Sectional	Crohn's disease, Ulcerative colitis	67	October 2005 – April 2007
Dannemann (2008) Germany [49]	Cross Sectional	T1DM	228	November 2004- December 2005
Day (2002) Australia [50]	Cross Sectional	Inflammatory Bowel Disease (IBD), Coeliac disease	92	April 2001
Day (2004) Australia [51]	Cross Sectional	Crohn's disease, Ulcerative colitis	46	January 2001-June 2002
Failing (2021) United States of America [52]	Cross Sectional	JIA	136	2017–2019
Feldman (2004) Canada [53]	Cross Sectional	JIA	118	Unspecified
Frawley (2017) Australia (13)	Cross Sectional	Autoimmune disease (non-specific)	149	Unspecified
Gerasimidis (2008) United Kingdom [54]	Cross Sectional	IBD	86	June 2005–2006
Gerkowicz (2020) Poland [55]	Cross Sectional	Crohn's disease, Ulcerative colitis	37	March-September 2019
Hagen (2003) Canada [56]	Cross Sectional	Arthritis (all subtypes), systemic lupus erythematosus, dermatomyositis, scleroderma, vasculitis, sarcoidosis, fibromyalgia	141	May–September 1999
Heuschkel (2002) United States of America [57]	Cross Sectional	Crohn's disease, Ulcerative colitis	208	January-October 2000
Hoffmann (2016) Canada [58]	Mixed Methods	Coeliac disease	145	Unspecified
Italia (2016) Germany [59]	Cross Sectional	Diabetes, Coeliac disease	4,677	January 2011 to October 2014
Kluthe (2018) Canada [60]	Qualitative	IBD—Crohn's disease, Ulcerative colitis	18	July 2015 to May 2016
Lemay (2011) Canada [61]	Cross Sectional	T1DM	195	Unspecified
Little (2019) United States of America [62]	Cross sectional	JIA	261	March 2016 -May 2017
Markowitz (2004) United States of America [63]	Cross Sectional	Crohn's disease, Ulcerative colitis	335	2000
Mattila (2013) Finland [64]	Cross Sectional	Coeliac disease	132	February 2007- May 2008
McCann (2006) United Kingdom [65]	Cross Sectional	IBD	75	Unspecified
McCarty (2010) United States of America [66]	Cross Sectional	Paediatric diabetes	467	January – August 2006
Miller (2008) United States of America [67]	Cross Sectional	T1DM	86	January- March 2004
Nousiainen (2014) Finland [68]	Cross Sectional	JIA, IBD	147	June 2011—June 2012
Pituch-Zdanowska (2019) Poland [69]	Cross Sectional	IBD—Crohn's disease, Ulcerative colitis	155	March 2016—March 2017
Rouster-Stevens (2008) United States of America [70]	Cross Sectional	JIA	52	June—July 2007
Samdup (2006) Canada [71]	Cross Sectional	T1DM	194	Unspecified
Sanchez (2011) United States of America [72]	Cross Sectional	JIA	132	Unspecified
Seburg (2012) United States of America [73]	Cross sectional	JIA	134	June – December 2010
Serpico (2016) United States of America [74]	Cross Sectional	Ulcerative Colitis, Crohn's disease, Indeterminate colitis	104	June – July 2014
Sgarlat (2011) United States of America [75]	Cross Sectional	JIA, Systemic Lupus Erythematosus, Mixed Connective Tissue Disease, Other rheumatologic diseases	202	June – October 2010
Southwood (2011) Australia, New Zealand, Canada [25]	Cross Sectional	Juvenile rheumatoid arthritis or juvenile chronic arthritis	53	Unspecified
Toupin-April (2009) Canada [76]	Cross Sectional	JIA	180	Unspecified
Vlieger (2008) Netherlands [77]	Cross Sectional	IBD, Coeliac disease	749	June 2003 – April 2004
Wadhera (2011) Australia [78]	Cross Sectional	IBD, Coeliac disease	97	October—December 2008
Wong (2009) United States of America [79]	Cross Sectional	IBD	362	2001—2003
Zebracki (2007) United States of America [80]	Cross Sectional	JIA	36	Unspecified

Regarding study design, cross sectional studies constituted 95% (*n* = 40), with only one mixed method and one qualitative paper included in the sample (refer to Table 2).

Inflammatory bowel disease (IBD), which includes ulcerative colitis and Crohn's disease, emerged as the most frequently documented paediatric AD managed with CAM, reported in eighteen primary research papers. Juvenile arthritic conditions, including rheumatological diseases, constituted the second most documented paediatric AD that was managed with CAM, cited in 16 primary research papers. This was followed by diabetes (*n* = 9) and Coeliac disease (*n* = 8) (Table 3).

**Table 3:** Types of autoimmune diseases in included studies

Types of Autoimmune diseases	Number of articles mentioned
IBD—Crohn's and Ulcerative colitis	18
Any arthritis or rheumatological autoimmune disease*	16
diabetes including T1DM	9
Coeliac	8
AD disease non specific	1
Other**	1

^*^rheumatological AD diseases include JIA, juvenile rheumatoid arthritis, systemic lupus erythematosus (SLE), scleroderma, vasculitis, fibromyalgia, mixed connective tissue disorder

^**^Other includes dermatomyositis, sarcoidosis

### Types of CAM used

The literature includes a diverse landscape of CAM modalities, which we have broadly classified into CAM products and CAM practices. There was variability in how CAM products were defined across papers. Typically, CAM products encompassed vitamins and minerals, herbal extracts, fish oil, probiotics, and other supplements. Vitamins and minerals had the highest reporting frequency (*n* = 25) (see Additional file 2). CAM practices included various therapeutic interventions such as homeopathy, traditional Chinese medicine, and faith healing. Among the extracted papers, massage (*n* = 20), homeopathy (*n* = 18), chiropractic care (*n* = 16), acupuncture (*n* = 16), dietary modifications (*n* = 14) and relaxation techniques (*n* = 13) were the most frequently reported practices, despite some papers intentionally excluding dietary modifications from their CAM definition (refer to Additional file 2).

The extraction of CAM usage data highlighted the inconsistency of how CAM use is measured in the literature. Data analysis revealed considerable variability among studies defining CAM and categorising specific practices or products under this umbrella. For example, Adams et al. [41] addressed the inconsistency regarding classifying vitamins and minerals as CAM. They incorporated both vitamins and minerals, including multivitamins, in their study. In contrast, Hagen et al. [56] considered self-prescribed single vitamins and/or minerals as CAM but excluded multivitamins. Markowitz et al. [63] omitted specific vitamins and minerals such as iron and folic acid but included megavitamin therapy (vitamins in supra-physiologic doses).

The variability in defining the different modalities constituting CAM was evident within and across different ADs. For instance, among the IBD population, Heuschkel et al. [57] and Markowitz et al. [63] excluded diet modifications from their definition of CAM, yet, Ceballos et al. [47] and Gerasimidis et al. [54] included them. Across ADs, diet modification was seen as an inclusive CAM practice in April et al.'s [43] study on the JIA population and Dannemann et al.'s [49] study on the Type 1 Diabetes Mellitus population. McCann and Newell [65] defined the inclusion and exclusion of CAM modalities in their study, reporting the exclusion of baby massage, simple vitamins and the use of aromatherapy oils for IBD. Inclusion of prayer as a CAM modality was also inconsistent in the literature. The National Centre for Complementary and Integrative Health’s (NCCIH) definition of CAM includes mindfulness and spiritual practices, encompassing prayer [18]. Heuschkel et al. [57] and Wong et al. [79] included prayer in their study of children with IBD and CAM use, whereas Markowitz et al. [63] and McCann and Newell [65] did not.

Furthermore, in the cross sectional studies, data on CAM were collected at various time points, ranging from baseline to different intervals post-diagnosis or initiation of the study. This variability in data collection methods poses a challenge in synthesising findings across studies. For instance, Adams et al. [41] assessed both lifetime and current CAM use, while Frawley et al. [13] examined CAM use over the past 12 months. April et al. [43] measured CAM use at baseline, 6-month, and 12-month intervals. Some studies provided specific time points for CAM use; for example, Hoffman et al. [58] investigated the duration of supplement use, ranging from less than one year to over ten years. In contrast, Heuschkel et al. [57] did not specify the time points of CAM use.

Population prevalence of CAM use could not be calculated from the extracted papers. The majority of studies recruited participants from subspecialty clinics, where a higher concentration of children with ADs was observed. For instance, Hagen et al. [56] included paediatric rheumatology patients attending an outpatient rheumatology clinic in a major tertiary care referral centre. Mattila et al. [64] recruited participants from the Finnish Celiac Society when investigating the burden of healthcare service use before and after Coeliac disease diagnosis in children, and McCann and Newell [65] included children attending specialist clinics at Leeds teaching hospitals who had a history of a chronic condition (IBD) for at least six months.

### Reasons for using CAM

Numerous studies examined the factors influencing families'use of CAM to manage their children’s AD. These factors have been categorised into two main categories; human agency, which reflects an individual's capacity to make decisions, and social structures, encompassing socio-demographic, structural, cultural, and economic influences [81]. Measuring the relative strength of these factors proved challenging due to the heterogeneous methodologies employed across studies. For example, while some studies, like Wadhera et al. [78], allowed respondents to provide reasons for CAM use through open-ended text, others, like Lemay et al. [61], documented the four most frequently cited reasons without specifying whether respondents could provide free-text responses or if CAM modalities were presented and then rated accordingly. Due to these methodological variations, reasons for CAM use, related to human agency and social structures, were documented based on their frequency of occurrence in the literature.

The distribution of papers exploring the reasons behind CAM use, encompassing both human agency and social structural factors, differed among various autoimmune conditions. The majority of literature examining reasons for CAM use was found in studies investigating inflammatory bowel disease (*n* = 13). Comparable numbers were identified in rheumatological conditions (*n* = 9) and diabetes (*n* = 8). Coeliac disease (*n* = 3) and AD in general (*n* = 1) had the fewest studies reporting on reasons for CAM use.

The large data extraction tables (Additional File 1, 3 and 4) contain a comprehensive list of reasons for CAM use, categorised by human agency and social structural factors. For brevity, similar reasons have been grouped together in Tables 4 and 5.

**Table 4:** Parental Reasons (individual level) why families utilised CAM to in manage their children's autoimmune diseases

Human Agency reasons parents use CAM for their children with an AD	Empirical Papers
Parental dissatisfaction with conventional medication	April et al. [43] Bode et al. [46] Day et al. [51] Dannemann et al. [49] Gerasimidis et al. [54] Heuschkel et al. [57] Samdup et al. [71] Sgarlat [75] Vlieger et al. [77] Wadhera et al. [78]
Parental concerns about side effects of conventional medications for children	April et al. [43] Bode et al. [46] Dannemann et al. [49] Day et al. [51] Failing et al. [52] Gerasimidis et al. [54] Heuschkel et al. [57] Lemay et al. [61] Vlieger et al. [77] Wong et al. [79]
Parental perception that CAM is safe and natural	Bode et al. [46] Dannemann et al. [49] Failing et al. [52] Gerasimidis et al. [54] Heuschkel et al. [57] Lemay et al. [61] McCann and Newell [65] Sgarlat [75] Vlieger et al. [77] Wadhera et al. [78]
Parental CAM use influencing child CAM use	Adams et al. [41] Adams et al. [42] April et al. [43] Ceballos et al. [47] Feldman et al. [53] Heuschkel et al. [57] McCann and Newell [65] Miller et al. [67] Sgarlat [75]
Parental belief that CAM will enhance overall child wellbeing	April et al. [43] Ball et al. [44] Bode et al. [46] Dannemann et al. [49] Miller et al. [67] Sgarlat [75] Vlieger et al. [77] Zebracki et al. [80]
Parental decision to incorporate CAM as a complementary therapy to conventional medicine for children	Day et al. [51] Gerasimidis et al. [54] Lemay et al. [61] Samdup et al. [71] McCann and Newell [65] Markowitz et al., [63]
Parental belief that CAM can induce remission of AD or provide hope for a cure	Dannemann et al. [49] Failing et al. [52] Heuschkel et al. [57] Lemay et al. [61] Sgarlat [75]
Parental decision to use CAM for alleviating children's pain	Failing et al. [52] Feldman et al. [53] Zebracki et al. [80]
Parental desperation to explore all options for children's AD treatment	Dannemann et al. [49] Day et al. [51] Sgarlat [75]
Parental decision to use CAM for child due to the perceived ineffectiveness of conventional treatments	April et al. [43] Vlieger et al. [77]
Parental decision to use CAM for a child is associated with the child’s vaccination status not being up to date	Frawley et al. [13]

**Table 5:** Structural (socio-demographic, economic and cultural) factors associated with CAM use

Structural Factors Associated with CAM use for children with an AD	Empirical Papers
Parental education	Parents with a Bachelor’s degree or higher were more likely to use CAM Frawley et al. [13] Gerkowicz et al. [55] Sgarlat [75] Wong et al. [79]
Family Income	Families in the income range of USD$50-100 K more likely to seek CAM Serpico et al. [74] Higher family income predictive of CAM use (no significant value provided) Gerkowicz et al. [55]
Disease duration	Children diagnosed at younger age more likely to use CAM Ceballos et al. [47] Longer disease duration associated with increased CAM use Feldman et al. [53] McCarty et al. [66]
Ethnicity	French Canadian parents are more likely to use CAM than English Canadian – Canadians in general more likely to use CAM than other ethnic groups Feldman et al. [53] The use of CAM was also more frequently reported for children whose parents were foreign born than for children whose parent was born in the United States Miller et al. [67]
Child age	Children aged younger than 11 years was a positive predictor for CAM use Vlieger et al. [77] Children aged 10 years or older had an increased use of CAM Sgarlat [75]
Parental age	Parents aged between 35-44 years were more likely to use CAM for their children Frawley et al. [13]
Geographical Location	Families from Urban areas more likely to use CAM Frawley et al. [13]
Marital status	Those married or in a de facto relationship more likely to use CAM for their children Frawley et al. [13]
Private Insurance	Those with private insurance are more likely to use CAM Serpico et al. [74]
Moderate to severe disease	Those with moderate to severe disease are more likely to use CAM than those with mild disease Serpico et al. [74]
School absenteeism, > 5 days	Greater use of CAM if child is away from school for more than 5 days Vlieger et al. [77]

#### Parental reasons CAM is used for children with an AD (human agency)

Table 4 outlines the eleven most frequently reported reasons for CAM use attributed to human agency. Among these, the most reported reasons include dissatisfaction with conventional medication, concerns regarding its side effects, and perceptions of CAM as natural or safer than conventional medicine. Nine studies found that parents who used CAM themselves were more likely to use CAM for their children with AD [41–43, 47, 53, 57, 65, 67, 75]. Additionally, eight studies reported parents felt CAM would enhance their child’s overall wellbeing [43, 44, 46, 49, 67, 75, 77, 80].

Interestingly, Frawley et al. [13] was the only paper to investigate and note an association between parents who used CAM for their children and their children’s vaccination status not being up to date.

#### Parental factors associated with CAM use for children with an AD (Structural)

Table 5 outlines the social structural factors linked to CAM usage. Notably, parents with at least a Bachelor's degree emerged as the demographic most likely to use CAM for their children with an AD. There were varying findings regarding the relationship between child age and CAM use. Vlieger et al. [77] identified children under 11 years old as more likely to use CAM, whereas Sgarlat [75] observed increased CAM use among children aged 10 years and older. Regarding ethnicity, Feldman et al. [53] reported higher CAM usage among children of foreign-born parents compared to those with U.S. born parents, while Miller et al. [67] noted a greater likelihood of CAM use among French Canadian parents compared to English Canadian parents, with Canadians in general more inclined toward CAM than other ethnic groups. Furthermore, Ceballos et al. [47] found an association between longer disease duration and increased CAM use. Similarly, Feldman et al. [53] found that children diagnosed at a younger age were more likely to use CAM.

### Experiences of parents using CAM

In the initial phases of the scoping review, it was anticipated that the literature would primarily yield qualitative research exploring the experiences of clients or families utilising CAM. However, upon full-text screening, it became apparent that most studies (40/42) employed cross sectional study designs, using surveys to capture data rather than qualitative methods such as interviews or focus groups. Consequently, the parental experiences of using CAM have been described from these cross sectional studies in the reasons for CAM use and the factors associated with CAM use sections of this paper. Data extraction provided further information classifiable as parental experiences of CAM use, such as the perceived benefits and/or efficacy of CAM use, adverse events and reasons for non-utilisation. This section of the paper will outline these aspects to continue to further describe parental experiences of CAM use.

Seven papers investigated the reasons for not using CAM [41, 42, 44, 51, 57, 58, 78]. Common reasons included concerns regarding the effectiveness of CAM therapy, cost, lack of knowledge about CAM therapy, effectiveness and registration of CAM therapists, and concerns regarding side effects when combined with other treatments.

Adverse events associated with CAM use were reported in six studies, with variations in measurement methodologies. Adams et al. [41] and [42] categorised adverse events by severity, with the majority classified as minor in both studies (69% and 57%, respectively). Additionally, Adams et al. [42] found that only 9.3% of parents reported adverse effects associated with CAM use. In contrast, Miller et al. [67] found that none of the respondents perceived CAM therapies as harmful. Similarly, Wadhera et al. [78] reported only one instance of CAM worsening the problem. Ball et al.'s [44] study found that only 2.9% of parents noted side effects from supplement use, including loose stools, nausea, vomiting, and stomach aches. Heuschkel et al.’s [57] study reported that 30% of patients experienced adverse effects from CAM usage, such as moodiness, weight gain, and headaches.

Eighteen papers examined the perceived benefits of using CAM. Among these, the three most commonly reported benefits were improvements in well-being, quality of life and disease state [30, 33, 43, 49–52, 55, 57, 61, 62, 66, 67, 70, 75, 77–79]. Miller et al.’s [67] study reported 81% of respondents found CAM therapies to be somewhat helpful; however, specific modalities were not specified. Sgarlat [75] found that 49% of CAM users in their study agreed that CAM therapy improved their child's disease state. They further categorised whole medicinal system practices as providing the most positive benefits (71%), followed by natural energy practices (63%) and natural products and manipulative practices (53% each). Similarly, Rouster-Stevens et al. [70] identified the CAM modalities users found most effective for children with JIA. These included support groups, spiritual healing, rubbing Vicks VapoRub® on the joints and vitamin D supplementation.

Disclosure of CAM use to conventional physicians was investigated across sixteen articles [13, 41, 42, 44, 49, 51, 52, 57, 61, 65, 67, 73, 75, 77, 78, 80]. The majority of these studies (69%) revealed that less than half of parents disclosed CAM use to their physicians [13, 41, 44, 51, 52, 57, 61, 65, 67, 73, 75, 80]. Reasons for non-disclosure included parents perceiving it as unimportant to inform their physicians, concerns about receiving negative responses from physicians or their physicians lacking knowledge about CAM, as well as some parents not having visited a physician since initiating CAM usage or physicians not asking about CAM usage [13, 44, 49, 52]. Conversely, a smaller proportion of studies (25%) reported that more than half of the parents disclosed CAM usage to their physicians [42, 49, 77, 78]. Danneman et al. [49] found that only a small fraction of physicians disapproved of CAM usage (9.2%) or displayed no interest (28.1%), while Vlieger et al. [77] reported that 54% of physicians reacted neutrally to the disclosure of CAM usage, 41% reacted positively, and 5% responded negatively.

## Discussion

This scoping review aimed to comprehensively assess all empirical research investigating how parents use CAM to manage their children’s AD. Its purpose was to provide CAM practitioners and researchers with baseline data on CAM usage among children with AD to support future clinical practice and research, offer biomedical professionals insights into the types of non-conventional therapies parents select and their motivations for doing so and inform parents about commonly utilised CAM therapies, empowering them to engage in informed discussions and openly disclose CAM practices to conventional medical practitioners. Findings indicate the most commonly reported reasons for CAM use attributed to human agency include dissatisfaction with conventional medication, concerns regarding its side effects, and perceptions of CAM as being natural or safer than conventional medicines. Parental use of CAM was predictive of child use, with CAM disclosure to conventional physicians remaining low. Reasons for non disclosure include parents reporting concerns about negative responses from physicians, perception of disclosure not being important or parents believing physicians do not have much knowledge about CAM.

Parental education emerged as the most commonly reported structural driver associated with CAM use, with the majority of parents possessing at least a Bachelor's degree or higher educational qualification. Additionally, family income emerged as another significant factor, with households earning between $50,000 and $100,000, or those with higher income levels, being predictive of CAM use. Notably, child age was the only structural factor that yielded varying results regarding CAM use.

It should be noted that human agency and structural factors likely influence each other within a dualistic system, as explained by Giddens' structuration theory [81]. Giddens argues that human agency encompasses the elements facilitating the creation of societal structures through the adoption of values, norms, or practices reinforced by social acceptance. Simultaneously, individuals are constrained by the existing social structure, unable to choose their parents, disease state, ethnicity, or the historical context in which they exist. This dual interaction between human agency and social structure highlights the complex interplay shaping individuals' behaviours and choices within society [82]. This suggests that societal structures may influence the decisions parents make regarding CAM use, just as individual human agency influences the shaping of these societal structures. Considering this sociological perspective, it is essential to recognise that social structure and human agency factors are interrelated. Future research should consider both structural and individual influences when investigating children’s CAM use in managing ADs.

### The most frequently reported (human agency) reasons parents are using CAM for their children with an autoimmune disease and disclosure to conventional physicians

The present study highlights dissatisfaction with conventional medication, concerns regarding its side effects, and perceptions of CAM as natural or safer than conventional medicines as the most frequently reported reasons parents use CAM for their children with AD. Additionally, Frawley et al. [13] found an association between lower vaccination rates for parents who used CAM for their children. Our findings align with childhood vaccination research, demonstrating parallels between families using CAM and those rejecting childhood vaccinations [83–85]. Investigations into vaccination rates among CAM-using families further support this association [86–88].

Attwell et al. [26] explore the concept of distrust in'expert systems,' encompassing government, healthcare professionals, and vaccine manufacturers. We propose that this distrust may underpin the dissatisfaction with conventional medicine, leading parents to seek CAM for their children with AD. Similarly, parents who opt out of vaccinating their children have reported feeling judged by conventional healthcare practitioners, potentially influencing their willingness to seek conventional care [89]. This sense of judgment may parallel the situation of CAM-using parents, contributing to low rates of CAM disclosure to their doctors. Our study reveals that parents often withhold information about CAM use from physicians due to concerns about negative responses, a perception that disclosure is inconsequential, or doubts regarding physicians' knowledge about CAM. This reluctance to disclose CAM use mirrors trends observed in the broader population of CAM users [90].

### Parental CAM use is associated with CAM use for children with autoimmune conditions

CAM is widespread within Australia's healthcare landscape, playing a significant role in the evolving consumer-driven healthcare sector [91]. Australian studies indicate that at least 63.1% of the general Australian population has used CAM, and over two thirds of parents are using CAM for their children under 18 years of age [11, 13]. Given the widespread use of CAM in Australia and the high rate of parental use preceding child use, it is conceivable that marketing may not significantly influence parental decisions to administer CAM to children with an AD. Instead, parental familiarity, positive experiences, and trust in CAM may drive their belief in its potential benefits for their children. Thus, CAM use for children with an AD appears to be influenced more by parental experiences and beliefs than by the influence of CAM therapists or marketing. As families have the autonomy to purchase registered or unregistered CAM products or CAM practices, questions arise about parental concerns regarding the safety and efficacy of these products. Parents cite CAM to be natural, equating it with being safe. This prompts questions: Do parents perceive themselves as serving a quality control role for their children's CAM use, or are they not concerned about potential side effects or the possible risks between the interplay of CAM products and conventional medicines? Do they feel more confident about CAM's safety and efficacy after personal experiences? Future studies could investigate why parental CAM use is the most commonly reported factor associated with CAM use for children with autoimmune conditions. Additionally, exploring whether CAM marketing has impacts on parental decision-making processes would provide valuable insights into how families navigate CAM within the healthcare landscape.

### Research designs

The majority of papers included in this scoping review employed cross sectional research methods. These cross sectional studies used surveys and questionnaires to collect data, limiting the ability of participants to offer open-ended responses about their use of CAM for their children with ADs. Interestingly, among the studies reviewed, those employing mixed-methods or qualitative approaches did not specifically investigate families’ experiences using CAM. This observation suggests an opportunity whereby future qualitative research can broaden our understanding of the ongoing and increasing use of CAM and its integration with conventional medicine for families. Exploring these nuanced family experiences, particularly through qualitative study designs, can offer deeper insights into the personal experiences in this context to help guide allopathic care, CAM care, and the overall experiences of clients and their families.

### Population prevalence

Population prevalence of CAM use could not be calculated from the extracted papers. The majority of papers drew participants from subspecialty clinics, where a higher concentration of children with ADs was observed. This sampling approach limits the generalisability of findings to the broader population, precluding comprehensive assessments of CAM utilisation trends at a population level. Future research should consider recruiting participants from the general population to provide more representative data on the prevalence of CAM use.

### Limitations of the review

#### How CAM use is measured and reported

The extraction of CAM usage data highlighted the inconsistency of how CAM use is measured and defined in the literature. Data analysis revealed considerable variability among studies defining CAM and categorising specific practices or products under this umbrella. Furthermore, different time points were utilised for data collection, ranging from baseline to various intervals post-diagnosis or initiation of the study, contributing to the challenge of synthesising findings across studies.

#### Only OECD countries included in the scoping review

This review is limited to studies from OECD countries, which may exclude important insights into CAM practices in non-OECD regions where traditional and alternative medicine play a significant role in healthcare [85]. As a result, widely used CAM modalities in these settings may not be captured, potentially limiting the applicability of findings to diverse global populations. However, this approach was chosen to align with the Australian healthcare context, ensuring relevance to its regulatory frameworks, socioeconomic factors, and clinical practices.

#### Search limited to English language

Due to the predominance of studies published in English, this review focuses on English-language literature, which may introduce a language bias and limit the comprehensiveness of the findings.

### Call to action:

The diverse range of CAM modalities noted in the literature highlights the multifaceted nature of CAM use among children with ADs. This review highlights the inconsistency in reporting its use and emphasises the pressing need for a professional body to establish standardised operating procedures. Such measures are essential to effectively gauge CAM use in a population, monitor changes over time, and implement interventions to promote its usage. Establishing baseline data through standardised protocols is needed to inform evidence-based strategies aimed at enhancing CAM use in paediatric AD management. This aligns with the findings of McCarty et al. [66] and Lee et al. [92] who similarly identify the need for future research to develop a reliable, validated and widely available assessment tool for CAM use in children and adults to facilitate comparison between studies. Additionally, Lee et al. [92, 93] highlight that standardising CAM definitions would further enhance the consistency and reliability of research findings in this field. Furthermore, future research into the reasons behind parents'use of CAM for children with ADs should consider the dualistic system comprising human agency and structural drivers of CAM use. These factors are interconnected components of a dualistic system that influences individuals' decisions. Investigating the interaction between these factors shaping CAM use is required for developing a deeper understanding of the complexities surrounding its use in managing ADs in children.

## Conclusions

This scoping review provides a comprehensive overview of empirical research on parental management of AD in children using CAM. The review identified various factors influencing CAM use, including both human agency and structural drivers. Dissatisfaction with conventional medication, concerns about side effects, and perceptions of CAM as natural or safer than conventional medicine were among the most frequently reported reasons for CAM use. Parental CAM use emerged as a significant predictor of CAM use for children with ADs, underscoring the influence of parental experiences and beliefs. However, low rates of CAM disclosure to conventional physicians suggest a need for improved communication and understanding between parents and healthcare providers.

Structural factors such as parental education, family income, and child age also played significant roles in CAM use. These findings highlight the complex interplay between individual decision-making and broader socio-economic influences on healthcare choices. Moreover, the scoping review identified methodological inconsistencies in measuring and reporting CAM use, emphasising the need for standardised protocols and increased awareness among researchers.

Moving forward, future research should consider the dualistic system comprising human agency and structural drivers when investigating CAM use in paediatric AD management. By understanding the interaction between these factors, healthcare professionals can better support families in making informed decisions about CAM use while ensuring the safety and efficacy of treatment approaches. Additionally, qualitative research exploring family experiences with CAM can provide valuable insights into the motivations, challenges, and outcomes associated with its use, improving patient-centred care and health outcomes for children with AD.

## Supplementary Information

Additional file 1. General information extracted from data files including lead author name, year and country of publication, title and aims of study, number of participants, reasons for using and for not using CAM, perceived benefits and efficacy of using CAM and any adverse events reported.

Additional file 2. Types of CAM used in different studies, including time points of CAM use if stated.

Additional file 3. Information about survey respondents including age, sex, overall health status, education, income, ethnicity, geographical location, and knowledge of CAM.

Additional file 4. Information about children including AD, child age, sex, disease severity, age of diagnosis, number of CAM therapies used, concurrent use of prescription medication and presence of other chronic illnesses.

Additional file 5. Search strategy used for database search.

## Data Availability

No datasets were generated or analysed during the current study.

## Abbreviations

AD: Autoimmune disease
CAM: Complementary and alternative medicine
T1DM: Type 1 diabetes Mellitus
JIA: Juvenile idiopathic arthritis
IBD: Inflammatory Bowel Disease
OECD: Organisation for Economic Co-operation and Development
PRISMA-ScR: Preferred Reporting Items for Systematic Reviews and Meta-Analyses extension for Scoping Reviews
IBD: Inflammatory bowel disease
NCCIH: National Centre for Complementary and Integrative Health
