# Supplementary material for: Parental management of autoimmune disease with complementary and alternative medicine: a scoping review of the literature in OECD countries
Source: BMC Complement Med Ther. 2025 Jul 10;25:255. doi: 10.1186/s12906-025-04929-4 (PMC12243366; doi:10.1186/s12906-025-04929-4)
Supplement: Supplementary file 5 — Additional file 5. Search strategy used for database search. [file 12906_2025_4929_MOESM5_ESM.docx]

### Additional File 5: Search strategy

Concept 1 (keywords)

("complementary medicine" OR "alternative medicine" OR "complementary and alternative healthcare" OR "complementary and alternative medicine" OR aromatherapy OR "natural medicine" OR yoga OR "herbal medicine" OR supplement* OR acupuncture OR naturopath* OR massage OR "complementary therap*" OR "holistic health" OR homeopath* OR "traditional medicine" OR phytotherap* OR "integrative medicine" OR "integrative health")

……………………………………………………………………………………………………………………………………………….

Concept 2 (keywords)

("autoimmune disease*" OR "autoimmune condition*" OR hashimoto* OR "Graves disease" OR "Coeliac disease" OR "celiac disease" OR "type-1 diabetes" OR "juvenile diabetes" OR "juvenile arthritis" OR "chronic disease" OR "chronic ill*" OR "inflammatory bowel disease")

……………………………………………………………………………………………………………………………………………

Concept 3 (keywords)

(child* OR adolescent* OR pediatric* OR paediatric*)

……………………………………………………………………………………………………………………………………………

Concept 4 (keywords)

(parent* OR caregive* OR mother* OR famili* OR mom* OR mum* OR father* OR dad*)
